# Mir-484 contributes to diminished ovarian reserve by regulating granulosa cell function via YAP1-mediated mitochondrial function and apoptosis

**DOI:** 10.7150/ijbs.68028

**Published:** 2022-01-01

**Authors:** Huiying Li, Xiaofei Wang, Hongbei Mu, Qiaojuan Mei, Yu Liu, Zou Min, Ling Zhang, Ping Su, Wenpei Xiang

**Affiliations:** 1 Institute of Reproductive Health, Tongji Medical College, Huazhong University of Science and Technology, Wuhan, Hubei 430030, China.; 2 Center of Reproductive Medicine, Tongji Medical College, Huazhong University of Science and Technology, Wuhan, Hubei 430030, China.

## Abstract

Women with diminished ovarian reserve (DOR) have reduced fertility, but the underlying regulation of ovarian function remains unknown. Although differential microRNA (miRNA) expression has been described in several ovarian disorders, little is known about the role of miRNAs in the pathogenesis of DOR. In this study, we investigated the expression levels of miR-484 in granulosa cells (GCs) derived from human follicular fluid, and explored their correlation with female ovarian reserve function as well as clinical outcomes of assisted reproduction technology (ART). Additionally, we investigated the effects of miR-484 on the biological functions of GC cell lines *in vitro*. We found that miR-484 was highly expressed in GCs from DOR patients and was correlated with decreasing AMH levels and AFC, as well as increasing FSH levels, but not with LH, progesterone, or estradiol. Additionally, miR-484 was negatively related to the number of retrieved oocytes and the ratio of high-quality embryos. Moreover, we found that miR-484 repressed the proliferation of GCs and induced apoptosis, which can in part be attributed to mitochondrial dysfunction. Conversely, silencing miR-484 had the opposite effect. Multiple approaches, including bioinformatic analysis, RNA-seq, qPCR, immunofluorescence, western blotting and luciferase reporter assays, identified YAP1 as a direct target of miR-484 in GCs. Additionally, reintroduction of YAP1 rescued the effects of miR-484 in GCs. The present study indicates that miR-484 can directly target the mRNA of YAP1, induce mitochondrial dysfunction, and consequently reduce the viability and promote the apoptosis of granulosa cells, which contributes to the pathogenesis of DOR.

## Introduction

Modern life has increased the number of factors causing infertility, including an unhealthy diet, sedentary lifestyle, environmental pollution, radiation and other stressors 1. The increasing incidence of infertility has brought tremendous pressure to clinical assisted reproduction 2. DOR is one of the main causes of female infertility 3. DOR is diagnosed in women of childbearing age that respond to ovarian stimulation or exhibited lower fertility than their peers, which is mainly manifested as a decrease in the number of follicles in the ovary and a decrease in the quality of oocytes 4. According to the Bologna criteria laid down by the European Society of Human Reproduction and Embryology, advanced age, abnormal ovarian reserve tests such as antral follicle count (AFC) and anti-Mullerian hormone (AMH), as well as prior suboptimal response to stimulation are the main diagnostic criteria for DOR 5.

GCs play an important role in the maintenance of ovarian function, and are considered the most important cell type in the ovary besides oocytes, affecting their development and maturation 6, 7. miRNAs are one of the most well-studied classes of small RNAs, which regulate more than half of human protein-coding genes 8, 9. Primary miRNAs are processed by Drosha to generate a precursor miRNA, which is further processed by Dicer to form the mature miRNA. Conditional knockout of the Dicer1 gene in ovarian GCs can cause ovarian dysfunction, including abnormal oocyte maturation, dysregulated ovarian follicular development and ovulation disorders, increased follicular atresia and even infertility 10. The function of GCs and cumulus cells can be affected by the increased or decreased expression of the many miRNAs related to the function of GCs, such as miR-145 targeting ACVR1b, or miR-92 and miR-21, which target SMAD7 to inhibit the apoptosis of GCs 11-13. Moreover, miR-205 and let-7g respectively target CREB1 and MAPK3K1 to promote the apoptosis of GCs and inhibit the synthesis of estrogen 14, 15. Recent studies have shown that miRNAs are differentially expressed in women with polycystic ovary syndrome and premature ovarian failure (POF) 16, 17. Research by Hong et al. has shown that plasma miR-23a levels are elevated in women with DOR and that treatment with pre-miR-23a induces apoptosis of cultured human GCs *in vitro* 18.

As an important regulatory factor, miR-484 is upregulated in many tumors and indicates a poor prognosis 19, 20. Recent research has shown that mi484 is related to mitochondrial fusion and division, and that it can suppress the translation of mitochondrial fission protein Fis1, thereby inhibiting Fis1-mediated fission in cardiomyocytes and adrenocortical cancer cells 21, 22. However, the role of miR-484 in the molecular pathogenesis of female fertility remains unknown, which prompted us to investigate its role in human GCs.

In the current study, we found that the expression of miR-484 was significantly increased in GCs from women with DOR, and its expression level was closely correlated with ovarian function and assisted reproductive technology. We demonstrate that upregulation of miR-484 decreases the levels of Yes-associated protein isoform 1 (YAP1) in human granulosa cells and induces apoptosis. YAP1 is the nuclear target of Hippo signaling whose overexpression can inhibit the expression of ERK and its downstream target dynamin-related protein 1 (Drp1). This in turn inhibits mitochondrial fission and rescues cardiomyocytes from mitochondrially triggered apoptosis 22. YAP1 might be another important player in DOR, which should be explored in future studies.

In the current study, we analyzed the expression levels of miR-484 in GCs from women with or without DOR. Compared with normal-cycling women, the levels of miR-484 in GCs from women with DOR were increased, which was accompanied by reduced YAP1 levels and impaired human granulosa cell function. The aim of this study was to further elucidate the role of miR-484 in the biological behavior of human GCs related to ovarian function, thus offering a better understanding of the molecular mechanisms underlying DOR.

## Materials and methods

### Sample collection and preparation

We collected follicular fluid samples from women undergoing oocyte retrieval for IVF/ICSI at the Center of Reproductive Medicine, Tongji Medical College, Huazhong University of Science and Technology between February 2018 and October 2019. Participants were required to meet the following requirements: 1) age between 20 to 45 years old; 2) unable to conceive naturally for at least 1 year, regardless of male, female, both, or uncertain factors; 3) primary or secondary infertility; and 4) IVF or ICSI cycles fertilized by husband or donor sperm. The mean age of the participants was 35.4 ± 9.3 years old (range from 20 to 45, n = 239). Participants with polycystic ovary syndrome, premature ovarian failure, endometriosis, and other reproductive endocrine diseases, such as thyroid disease, diabetes, adrenal disease, etc., were excluded. The basic information of the patients (n = 114) who qualified for the final analysis is presented in Table 1. This study was approved by the ethics committee of Tongji Medical College, Huazhong University of Science and Technology (Approval. No. 2018 (02); February 25, 2018, Wuhan, China).

### Isolation, purification and identification of GCs

On the day of oocyte retrieval, all follicular fluid samples from the same patient were pooled after cumulus-oocyte complexes were isolated for conventional *in vitro* fertilization (IVF) or intracytoplasmic sperm injection (ICSI) procedures. GCs were isolated from follicular fluid following the standard operating procedure. First, the follicular fluid was gently placed over 50% of Percoll solution (P8370, Solarbio, Beijing, China). After centrifugation at 1500 rpm/min for 10 minutes, the solution was divided into 3 layers. Cells in the middle layer were collected with a Pasteur pipette. The GCs were obtained by treatment with blood cell lysis solution (R1010, Solarbio, Beijing, China) to lyse erythrocytes, after which the GCs were washed with PBS. The purity of the GCs was confirmed using previously described methods 23. Follicle stimulating hormone receptor (FSHR) was selected as a granulosa cell specific marker to assess the cell purity. GCs were stained with antibodies against FSHR (1: 100; ab113421, Abcam, Cambridge, MA, USA) according to a previous study. These harvested GCs were stored in TRIzol reagent (Vazyme, China) at -80 °C until RNA extraction.

### Cell culture and treatment

The GC cell line COV434 was purchased Shanghai Honsun Biological Technology Co., Ltd, which obtained the cell line from the American Type Culture Collection (ATCC). The GC cell lines were maintained in Dulbecco's modified Eagle's medium (DMEM) with 10% fetal bovine serum (Gibco, USA) and 1% penicillin/streptomycin (Gibco, USA) at 37 °C in an atmosphere comprising 5% CO_2_, and stored in FBS with 10% DMSO at -80°C. Cells were transfected with 100 nM miR-484 mimic or miR-484 inhibitor as well as their respective negative controls (RiboBio, China) using Lipofectamine 3000 (Invitrogen, USA). si-YAP1 was purchased from RiboBio. The sequences used are provided as follows: si-YAP1: 5'- CCACCAAGCTAGATAAAGA -3'. The YAP1 overexpression plasmid and its negative control were purchased from Genomeditech Co., Ltd (Shanghai, China).

### RNA-sequencing

Total RNA was quantified using a NanoDrop 2000 spectrophotometer (NanoDrop Technologies, Wilmington, DE, USA). COV434 cells were collected 48 h after transfection with 100nM miR-484 mimic and/or negative control (RiboBio, China). Whole-exome RNA-sequencing was performed by Annoroad Gene Technology (China) according to a standard procedure.

### Isolation of mouse GCs and mouse cumulus cells (CCs)

Mouse GCs and CCs were collected from the ovaries of 23- to 26-day-old mice, following induction of superovulation by intraperitoneal injection of PMSG (Pregnant Mare Serum Gonadotropin, Solarbio, China). After approximately 48 hours, the animals were euthanized by cervical dislocation, and the ovaries were removed and placed into DMEM. Then, the ovarian follicles were punctured with a needle under a stereomicroscope. The released cells were scattered and washed in the DMEM, after which the cellular debris and oocytes were filtered through a cell sieve (NEST, China). The filtered GCs and CCs were collected into a 15 ml centrifuge tube and centrifuged at 400g for 5 min. Then, the supernatant was discarded, after which the mixed cells were twice washed with serum-free medium and cultured in a 24-well plate. GCs were cultured in DMEM (Gibco, USA) supplemented with 10% FBS and 1% penicillin-streptomycin. All the animal experiments were authorized by Institutional Animal Care and Use Committee of Tongji Medical College, Huazhong University of Science and Technology (IACUC number: S2061).

### Fluorescence *in situ* hybridization (FISH)

*In situ* hybridization was performed using a commercial FISH Kit (RiboBio, China). Briefly, GCs and CCs grown on the slides were washed with PBS and fixed in 4% paraformaldehyde. After protease treatment, the slides were incubated with pre-hybridization buffer at 40 °C for 4 h, and then hybridized with a digoxin-labeled probe at 40 °C overnight. After washing and blocking, the slides were incubated with a biotin-conjugated anti-digoxin antibody. The slides were then washed and incubated with SABC-FITC at 37 °C for 30 min. The images were captured using a confocal microscope (Carl Zeiss, Germany).

### Cell viability assay

Cell viability was determined using the Cell Counting Kit-8 assay (Vazyme, China). Briefly, 10 μl of the kit reagent was added to each well of a 96-well plate and incubated at 37 °C for 1 h. The spectrophotometric absorbance at 450 nm of each sample was measured using a Gen5 Multifunctional Microplate Reader (BioTek, USA).

### Quantitative real-time PCR (qRT-PCR)

Total RNA was extracted using Trizol (Vazyme, China) and reverse-transcribed using random primers with the PrimeScript RT reagent kit (Vazyme, China). The qRT-PCR was performed on a Quantagene q225 real-time PCR system (Kubo, China) using the AceQ qPCR SYBR Green Master Mix (Vazyme, China), according to the manufacturer's instructions (95°C for 3 min and 40 cycles of 95 °C for 10 s, annealing temperature (58-62 °C) for 30 s, and 72 °C for 30 s, followed by 72 °C for 1 min). The relative expression of candidate miRNAs was normalized to U6 and then calculated using the 2^-ΔΔCt^ method. All experiments were repeated in triplicate and presented as the means ± SD. The primer sequences are listed in Supplementary Table 1.

### Western blotting

Total protein from the GCs was extracted using RIPA reagent (Servicebio, China), denatured, separated on a 12% polyacrylamide SDS-PAGE gel, and transferred to a nitrocellulose membrane. After blocking, the blots were incubated with antibodies against YAP1 (1:1000, 14074S, CST, USA) and β-actin (1:1000, 66009-1-Ig, Proteintech, China) in Tris-buffered saline (TBS) containing 0.1% Tween-20 at 4 °C, overnight. The following day, the blots were incubated with a goat anti-rabbit or anti-mouse horseradish peroxidase immunoglobulin G (H+L) secondary antibody (1:5000, SA00001-2, Proteintech, China) at 37 °C for 2 h. The protein bands were detected using a chemiluminescence detection kit (Servicebio, China) on X-ray films and analyzed using Quantity One software (Bio-Rad, USA).

### Luciferase reporter assay

The 3'-UTR of YAP1 contains conserved miR-484 binding sites. Mutated 3'-UTR of YAP1 were synthesized using a QuikChange II XL Site-Directed Mutagenesis Kit (Stratagene), and the fragment was amplified by PCR. HEK-293 cells were co-transfected with the 3'-UTR luciferase vector and miR-484 mimics using Lipofectamine 3000 (Invitrogen, USA). Renilla luciferase was used as internal reference. After 48 h of transfection, luciferase activities were measured using a Dual-Luciferase Reporter Assay System (Promega) following the manufacturer's instructions.

### Flow cytometry

Annexin V FITC/PI staining was performed to detect apoptotic cells. First, transfected cells were washed 3 times with ice-cold PBS, resuspended in 100 µl of binding buffer, followed by the addition of 5 µl Annexin V-FITC (20 mg/mL) and 10 µl PI (50 mg/mL) (Beyotime, China). After incubation at room temperature for 15 min, 400 µL of binding buffer was added and the cells were injected into a flow cytometry instrument (Invitrogen, CA, USA). The cell cycle status was determined using a PI staining assay (Sigma, USA). Briefly, cells were subsequently fixed with 70% ethanol at 4 °C for 12 h. The fixed cells were then stained with 500 μL PI/Triton X-100 solution (0.02% RNAse, 0.002% PI, 0.1%, Triton X-100) at 37 °C for 15 min. And data was acquired using BD Accuri Cytometry (BD BioSciences, Franklin Lakes, NJ, USA).

### Fluorescence staining

A mitochondrial superoxide indicator (Beyotime, China) was used to quantify mitochondrial ROS (mtROS) production. The cells were rinsed and incubated with the fluorescent probe in the dark at 37 °C for 25 min. The mtROS content was expressed as the area of intensity (AOI). Mitochondrial membrane potential (MMP) depolarization was assessed using a JC-1 fluorescent probe and was represented as the ratio of red to green fluorescence intensity.

### Extraction and quantification of mtDNA from GCs

mtDNA was extracted from GCs using the TIANamp Genomic DNA kit (TIANGEN, China). The copy number and concentration of mtDNA was estimated via amplification using ND1 and β-globin primers and real-time qPCR. The sequences are listed in Supplementary Table 1.

### Measurement of ATP levels in GCs

The relative ATP content was measured using an ATP Assay Kit (Beyotime, China). The cells were lysed with ATP lysis buffer and centrifuged at 12,000 g and 4 °C for 5 minutes. The working solution was added to the opaque 96-well plate first, after which the sample was added to the detection well, and the relative light unit (RLU) value was measured using a luminescence microplate reader (Awareness Technology, Inc., USA).

### Statistical analysis

Statistical analyses were performed using SPSS Statistics (version 23.0; IBM, Armonk, NY, USA) and GraphPad Prism 8.0 (version 8.0c; GraphPad Software, Inc., San Diego, CA, USA). All of the data are displayed as the mean ± SD for triplicate independent measurements. One-way ANOVA was used to assess the differences between groups. Differences with P values < 0.05 were considered statistically significant.

## Results

### Baseline information of the participants

We collected 156 samples of human follicular fluid. After extracting GCs by Percoll density gradient centrifugation, a total of 114 women were enrolled in this study. The inclusion and exclusion criteria are shown in the materials and methods. The participants' demographics and baseline characteristics, including age, BMI, duration of infertility, baseline female hormone levels, antral follicle count (AFC) and anti-Mullerian hormone (AMH) levels are presented in Table 1. GCs are the only cells expressing FSH receptor *in vivo*. Therefore, anti-FSHR immunofluorescence staining was used to identify GCs in human follicular fluid (Fig. S1A). This is a gold standard method that is generally used for assessing the purity of granulosa cells. It was found that almost 100% of the GCs used in our experiment expressed this specific marker, which confirmed the purity of GCs.

### miR-484 expression levels in GCs from human follicular fluid are associated with ovarian reserve and assisted reproductive technology outcomes

We validated the general biological characteristics of miR-484. As the results demonstrated, miR-484 could be amplified from GCs *in vitro* using specific primers (Fig. S1B). These collected samples were divided into a young group (≤35 years old, n=65) and aging group (>35 years old, n=17) according to the age of the probands. According to ovarian reserve, the probands were divided into a normal ovarian reserve group (NOR, n=65) and diminished ovarian reserve group (DOR, n=23). The ovarian reserve status was assessed according to the Bologna criteria laid down by the European Society of Human Reproduction and Embryology.

**Figure 1:**
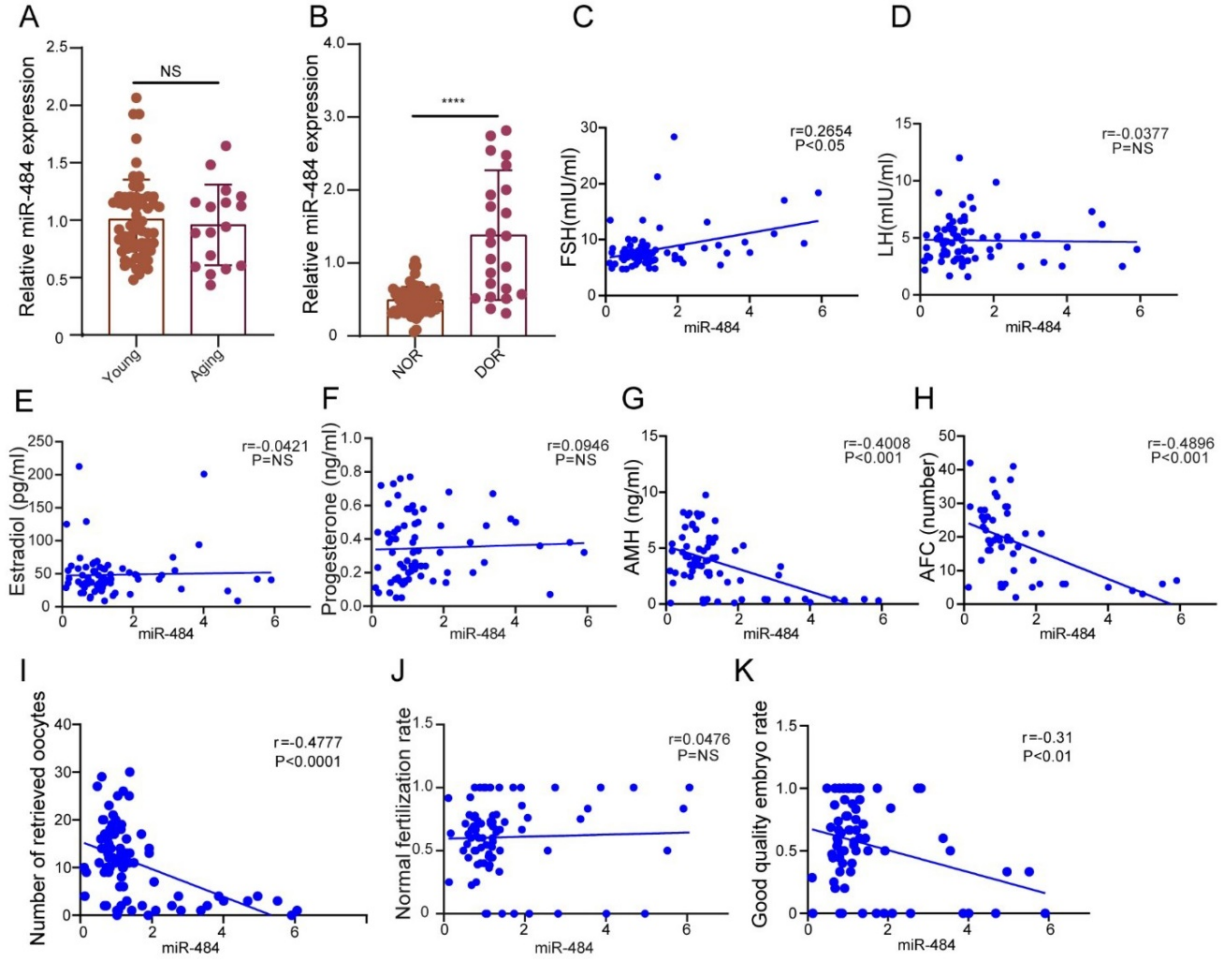
** The expression level of miR-484 in GCs from human follicular fluid is associated with the ovarian reserve and assisted reproductive technology outcomes. (A-B)** The relative expression levels of miR-484 in females of different age and different ovarian reserve groups. **(C-H)** Correlations between the levels of miR-484 and reproductive hormones (FSH, LH, estradiol, progesterone, AMH) and antral follicle counts (AFC). **(I, J, K)** Correlation analysis of the expression level of miR-484 with the number of retrieved oocytes, normal fertilization rate and ratio of high-quality embryos. According to the consensus of experts of the Society for Assisted Reproductive Technology (SART), the normal fertilization rate was calculated as the number of 2PN/number of fertilized oocytes; the ratio of high-quality embryos was calculated as the number of high-quality embryos/number of 2PN embryos. Each bar in the figure represents the mean ±SD. Young group (≤35 years old, n=65) and Aging group (>35 years old, n=17). Normal ovarian reserve group (NOR, n=65) and Diminished ovarian reserve group (DOR, n=23). NS, not statistically significant; *, P < 0.05; **, P < 0.01; ***, P < 0.001; ****, P < 0.0001.

The expression level of miR-484 in GCs from human follicular fluid was significantly higher in the DOR group than in the NOR group (P < 0.0001, Fig. 1B), whereas no significant age-related differences were found (P > 0.05, Fig. 1A). The expression level of miR-484 in GCs was positively correlated with the basal FSH level (r=0.2654, P<0.05, Fig. 1C). Further analysis demonstrated that there was no remarkable correlation between the level of miR-484 in GCs from human follicular fluid and LH, estradiol or progesterone (both P > 0.05, see in Figs. 1D, 1E​, and 1F). However, it was negatively correlated with AMH (r=-0.4008, P<0.001, Fig. 1G) and antral follicle count (AFC) (r=-0.4096, P<0.001, Fig. 1H).

Considering the ART outcomes, we analyzed the correlation between the expression of miR-484 in GCs and the number of retrieved oocytes, the normal fertilization rate and the ratio of high-quality embryos. The results indicated that the expression level of miR-484 was negatively correlated with the number of retrieved oocytes (r=-0.4777, P<0.0001, Fig. 1I) and the ratio of high-quality embryos (r=-0.31, P<0.01, Fig. 1K). However, there was no significant linear relationship between the expression level of miR-484 and the normal fertilization rate of oocytes (Fig. 1J).

### Identification of miR-484 in GCs

Here, the expression of miR-484 was measured in various tissues of mice, and the results indicated that miR-484 is expressed in various tissues (Fig. S1C). At the same time, the relative expression level of miR-484 in the ovaries of mice was quantified at several key time points during development, and the changes in the expression level of miR-484 in the ovaries from newborn to mature mice were systematically analyzed. The results indicated that miR-484 may play a regulatory role in the normal development of mouse ovaries (Fig. S1D). The nucleotide sequence of the miR-484 precursors is highly conserved in mammals (Fig. S1E). We also identified five asymmetric bulges in the structures of the hsa-miR-484 duplexes (Fig. S1F). In addition, we analyzed the expression pattern of miR-484 in a variety of GCs, which revealed that miR-484 was localized to the cytoplasm and nucleus of human GCs, mouse GCs and mouse cumulus cells (Figs. S1G-I).

### MiR-484 compromises GC function by influencing mitochondrial function and apoptosis

**Figure 2:**
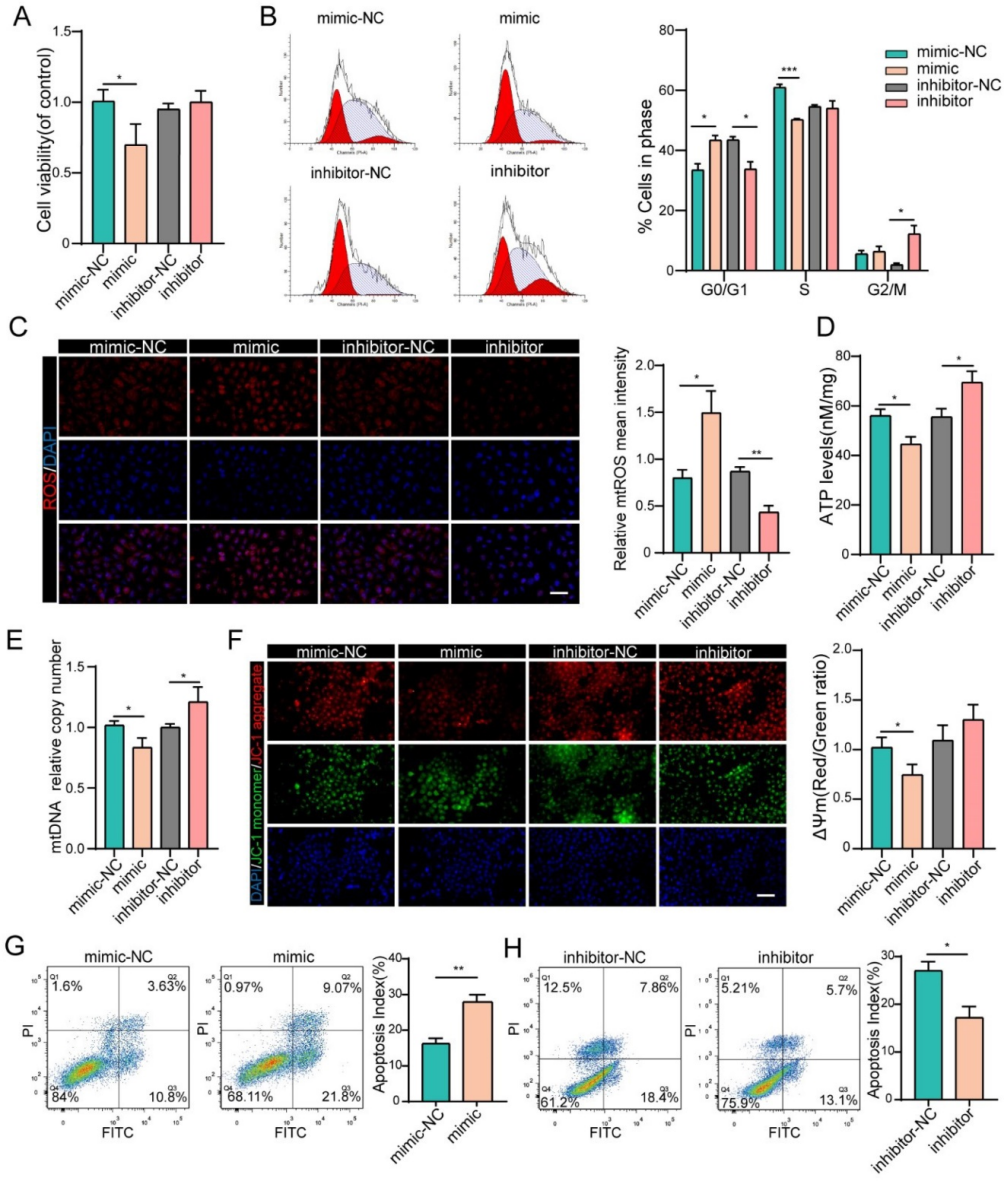
** miR-484 affects GC function by regulating mitochondrial function and related apoptosis. (A-B)** Cell viability changes and cell cycle alterations were quantified. mtROS (red) in GCs were measured and quantified **(C)**. **(D-E)** ATP and mtDNA levels were measured. **(F)** The mitochondrial membrane potential (ΔΨm) was measured using the JC-1 assay. **(G-H)** Apoptotic cells were detected by flow cytometry. Bar graphs illustrating the percentage of viable (Q3), early apoptotic (Q4), late apoptotic (Q2), and necrotic (Q1) cells according to Annexin V FITC/PI staining. Each bar in the figure represents the mean ± SD from three replicates. Bar =50 µm. *, P < 0.05; **, P < 0.01; ***, P < 0.001 compared with the NC group.

We next investigated the effects of miR-484 on GC function via loss- and gain-of-function experiments. We confirmed the identity and purity of the granulosa cell line COV434 using antibodies against FSHR (Fig. S1A). Additionally, miR-484 was overexpressed or silenced in GCs using a miRNA mimic/inhibitor (Figs. S2A and B). The overexpression of miR-484 obviously reduced the cell viability and proliferation ability (Fig. 2A). To assess whether miR-484 influenced the cell cycle progression of GCs, the cell cycle distribution was analyzed by flow cytometry. An obvious increase in the number of cells in the G0/G1 phase was observed by ModFit analysis, suggesting that overexpression of miR-484 increased the abundance of cells in the G0/G1-phase (Fig. 2B). Additionally, miR-484 overexpression increased oxidative stress, as shown by the enhanced mitochondrial ROS (Fig. 2C). Conversely, downregulating miR-484 had the opposite effect. Next, we focused on the effects of miR-484 on mitochondria by measuring the ATP content and mtDNA expression levels. These results indicated that mitochondrial ATP levels and mtDNA levels of GCs were decreased after transfection with miR-484 mimic, suggesting that miR-484 can affect mitochondrial and cellular functions (Fig. 2D,2E). It seems that miR-484-overexpressing GCs had a reduced ability of both mitochondrial ROS scavenging and ATP synthesis, which was likely associated with the loss of mitochondrial function. However, the miR-484 mimic obviously enhanced apoptosis by activating the mitochondrial pathway, as indicated by the markedly increased mitochondrial membrane potential (MMP) depolarization (Fig. 2F). Furthermore, flow cytometry showed that miR-484 mimic significantly decreased the percentage of viable cells and increased the percentage of early and late apoptotic cells (Fig. 2G). Conversely, the opposite results were obtained when miR-484 was inhibited (Figs. 2F and H).

### The effects of miR-484 on GCs on a transcriptome-wide scale

**Figure 3:**
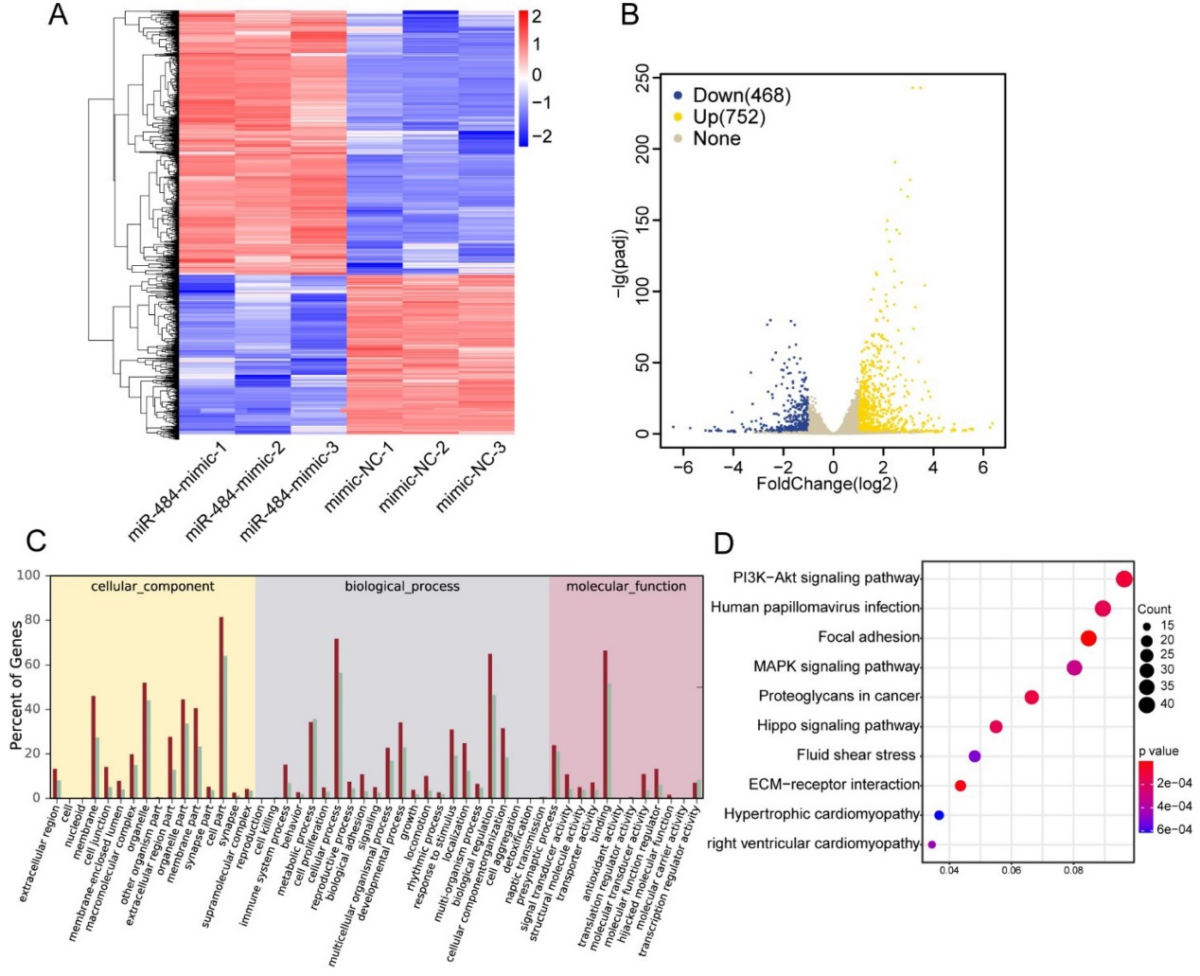
** Effects of miR-484 on GCs on a transcriptome-wide scale. (A)** Heatmap of the DEGs in GCs overexpressing miR-484 according to RNA-seq. **(B)** Scatter chart of all the expressed genes in GCs overexpressing miR-484. **(C)** Gene Ontology analysis of transcripts from GCs overexpressing miR-484. **(D)** Pathway analysis of transcripts from GCs overexpressing miR-484.

To further identify the associated pathways and direct targets of miR-484 in GCs, RNA-seq was performed in miR-484-overexpressing GCs. To determine the effect of miR-484 mimics on the genome-wide transcriptional activity, we performed comparative transcriptome analysis, with GC cells expressing mimics as control (Fig. 3A). A total of 26,432 genes were analyzed, and 1220 genes were differentially expressed according to a 2-fold difference as the cut-off value (Benjamini-Hochberg adjusted P-value < 0.05). A heatmap was used to visualize differential gene expression (Fig. 3A). We observed that overexpression of miR-484 led to the downregulation of 468 genes and upregulation of 752 genes (Fig. 3B). We also performed GO analysis on the dysregulated genes in cells expressing miR-484 and found that miR-484 participates in different biological events and plays different roles by regulating target genes (Fig. 3C). The dot-plot of KEGG pathway analysis in Fig. 3D shows the results of the top 10 pathway according to the P value, from small to large. Additionally, signaling pathway analysis showed that the hippo pathway was a potential candidate pathway. The expression levels of several genes from the hippo signaling pathway are shown in Fig. S2C. Some essential genes in this pathway, including ID1, PARD6A, ID2, YAP1, and SMAD1, were significantly repressed by miR-484 in GCs (Fig. S2C). These results suggest that miR-484 and the hippo signaling pathway might form a negative feedback loop.

### YAP1 is a direct target of miR-484 in GCs

**Figure 4:**
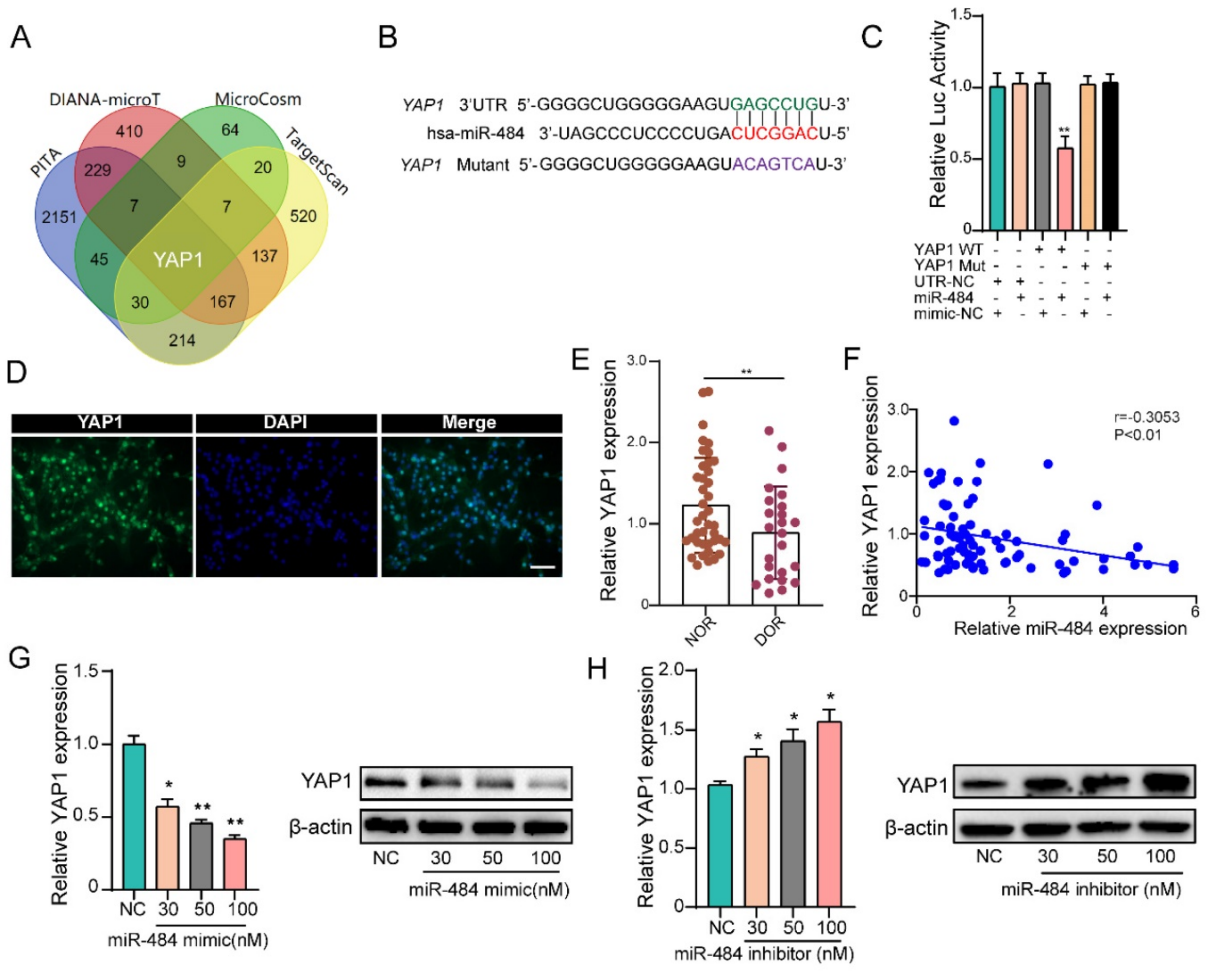
** YAP1 is a direct target of miR-484 in GCs. (A)** Predicted targets of miR-484 according to four algorithms. **(B)** The miR-484 binding site in the 3'UTR of YAP1 was predicted using TargetScan. **(C)** The luciferase reporter assay showed direct binding between the miR-484 and the YAP1 3' UTR. **(D)** Expression and localization of YAP1 in human GCs. **(E-F)** GCs were transfected with increasing amounts of the miR-484 mimic and inhibitor, followed by quantitative analysis of changes in the mRNA and protein expression of YAP1. Bar: 25 µm. *, *P* < 0.05; **, *P* < 0.01; ***, *P* < 0.001 compared with the si‐NC group.

By combining the RNA-seq and miRNA target prediction software results, YAP1 was identified as a potential direct target of miR-484 in GCs (Fig. 4A). TargetScan indicated that 3'UTR of YAP1 contains a potential miR-484 seed sequence match (Fig. 4B). To determine whether YAP1 was a direct target of miR-484, the putative miR-484 target in the 3' UTR was cloned into a reporter plasmid downstream of the luciferase coding sequence. The results showed that miR-484 mimics repressed the fluorescence from the 3' UTR, indicating that miR-484 could directly bind to the 3' UTR of YAP1 (Fig. 4C). Moreover, we detected the expression and localization of YAP1 in human GCs (Fig.4D). The expression level of YAP1 in GCs from human follicular fluids in DOR group was significantly lower than that in NOR group (P < 0.01, Figure 4E). In addition, correlation analysis suggested a negative correlation between the level of miR-484 and YAP1 expression level in GCs (Fig. 4F). Then, GCs were transfected with different concentrations of the miR-484 mimic or inhibitor to explore whether miR-484 could modulate YAP1 transcription and translation. As shown in Fig. 4G, both the mRNA and protein expression of YAP1 was obviously reduced in a dose-dependent manner after miR-484 mimic transfection. By contrast, transfecting the cells with increasing concentrations of the miR-484 inhibitor gradually promoted YAP1 expression (Fig. 4H). These results confirmed that miR-484 could directly bind to the mRNA of YAP1 in GCs and negatively control its transcription.

### YAP1 positively regulated GC function

**Figure 5:**
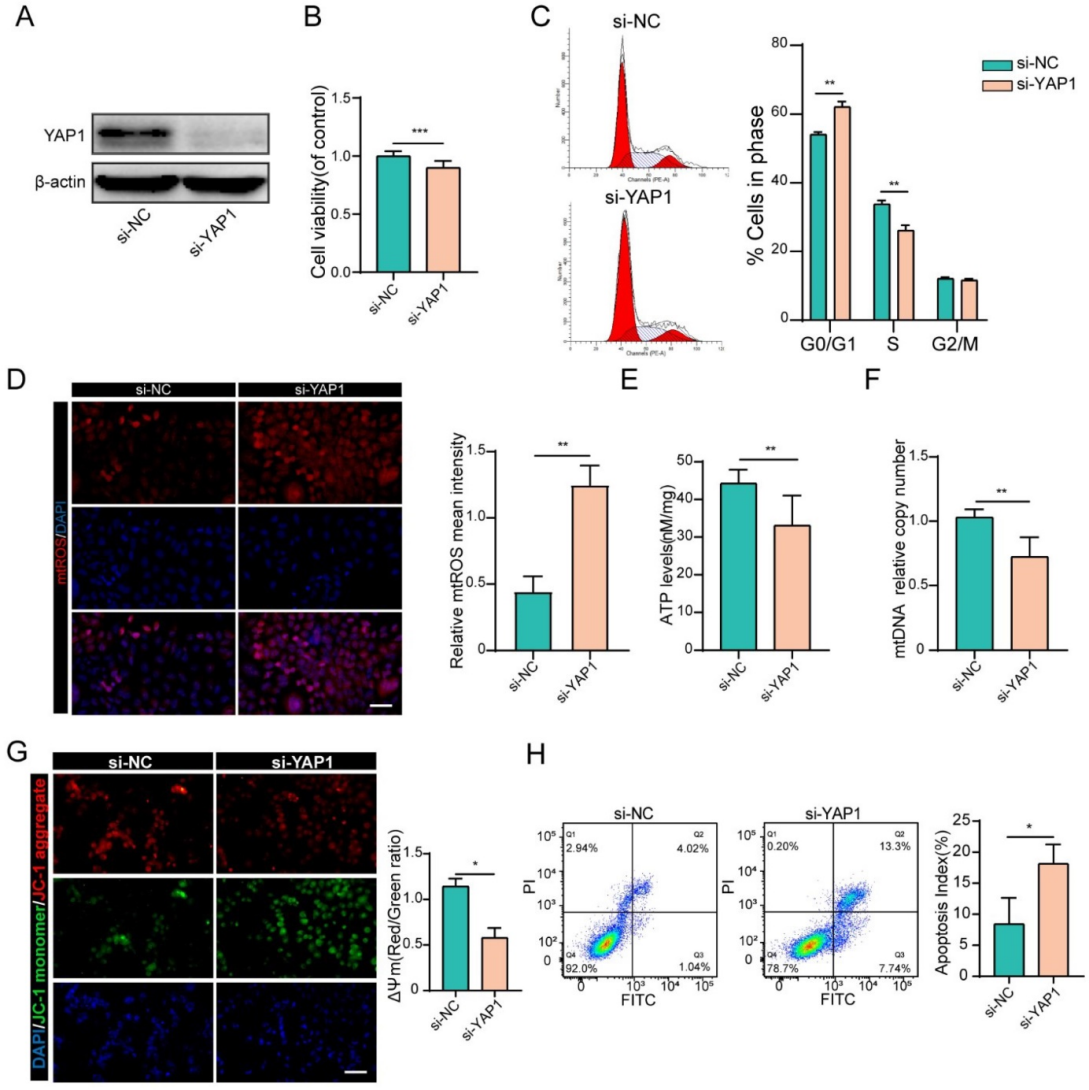
** YAP1 positively regulated GC functions. (A)** Transfection effectively knocked down YAP1 expression. **(B)** Proliferation was inhibited by YAP1 knockdown in GCs. **(C)** cell cycle alterations were analyzed. **(D)** mtROS (red) in GCs were measured and quantified. **(E-F)** ATP and mtDNA levels were measured following YAP1 knockdown in GCs. **(G)** The mitochondrial membrane potential (ΔΨm) was measured using the JC-1 assay. **(H)** Flow cytometry showed that apoptosis was induced by YAP1 knockdown in GCs. Each bar in the figure represents the mean ± SD from three replicates. Bar: 50 µm. *, *P* < 0.05; **, *P* < 0.01; ***, *P* < 0.001 compared with the si‐NC group.

To prove that YAP1 acts as the downstream target of miR-484 that mediates its function in GCs, siRNA was used to knock down YAP1 (Figs. S2D and E). As shown in Fig. 5A, si-YAP1 had an evident knockdown effect and was chosen for the subsequent experiments. CCK-8 assays demonstrated that silencing YAP1 significantly reduced the proliferation of GCs (Fig. 5B). Compared with cells in the si-NC group, there was an obvious increase in the abundance of cells in the G0/G1 phase in the si-YAP1 group, suggesting that YAP1 knockdown induced G0/G1-phase arrest (Fig. 5C). As expected, GCs transfected with si-YAP1 presented higher oxidative stress injury, which was reflected in higher mtROS levels (Fig. 5D). Similar to the GCs overexpressing miR-484, the ATP content and mtDNA levels of GCs transfected with si-YAP1 were significantly decreased (Fig. 5E, 5F). Moreover, silencing YAP1 obviously aggravated the MMP depolarization in GCs (Fig. 5G), and flow cytometry showed a significant decrease in the percentage of viable cells and an increase in the percentage of early and late apoptotic cells among the YAP1-deficient GCs (Fig. 5H). These results were consistent with the phenotype of GCs overexpressing miR-484, which suggest that the antiproliferative and proapoptotic effects of miR-484 in GCs may be mediated by reduced YAP1 expression.

### YAP1 rescues miR-484-induced mitochondrial damage and apoptosis in GCs

**Figure 6:**
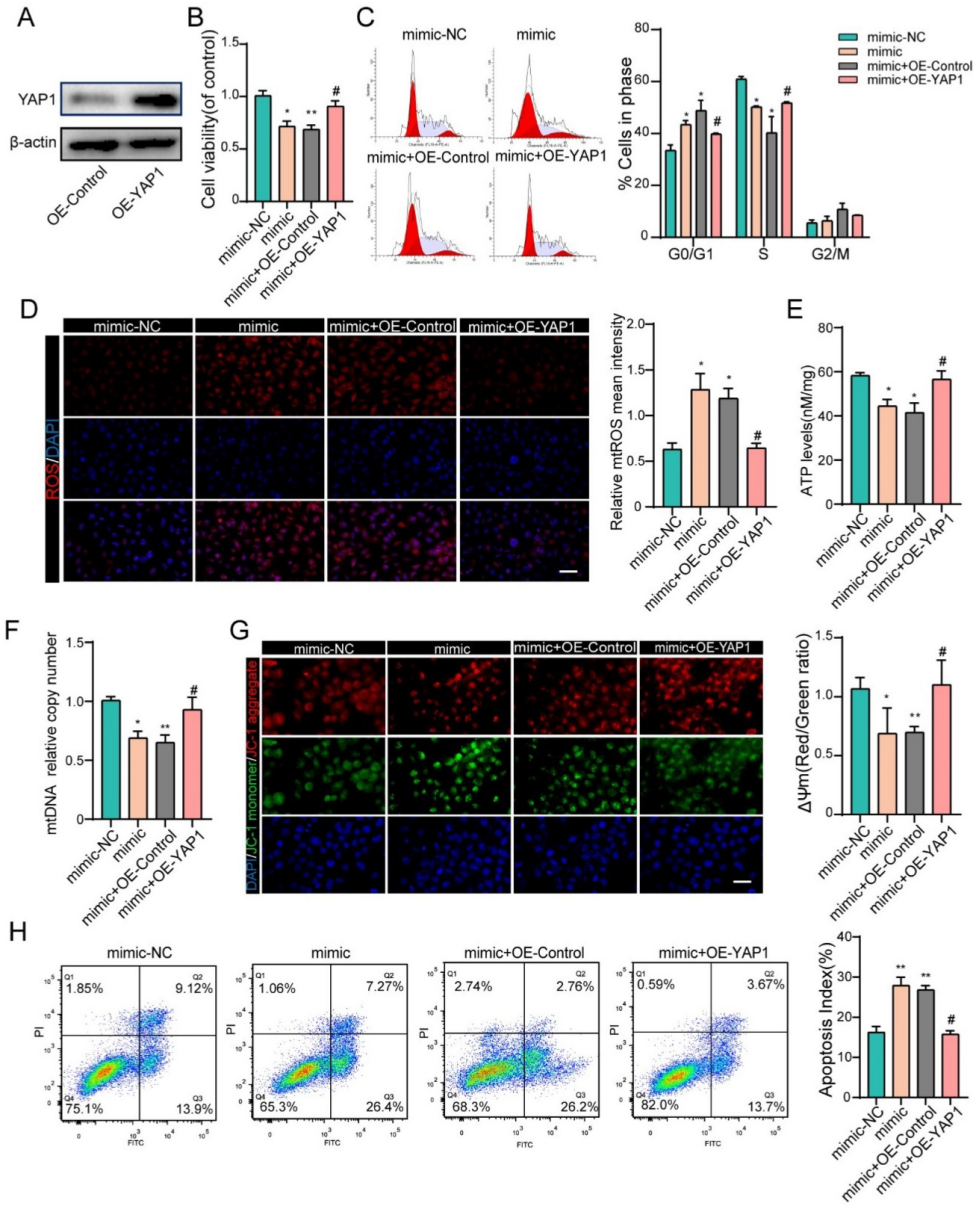
** YAP1 rescued the effects of miR-484 in GCs. (A)** Western blot showing that YAP1 was overexpressed in GCs. **(B)** YAP1 rescued the miR-484-induced repression of proliferation in GCs. **(C)** Cell cycle distribution was analyzed using flow cytometry. **(D)** mtROS (red) in GCs were measured and quantified. **(E-F)** ATP and mtDNA levels were measured. **(G)** The mitochondrial membrane potential (ΔΨm) was measured using the JC-1 assay. **(H)** YAP1 rescued miR-484-induced apoptosis in GCs. Each bar in the figure represents the mean ± SD from three replicates. Bar: 50 µm. *, *P* < 0.05; **, *P* < 0.01; ***, *P* < 0.001 compared with the miR‐NC group, #*p* < 0.05 compared with the mimic+ OE-NC group.

To further confirm that the negative effects of miR-484 in GCs are at least partially mediated by YAP1 downregulation, recombinant YAP1 was used to treat miR-484-overexpressing GCs. We first overexpressed YAP1 in GCs and verified the overexpression efficiency by western blotting and immunofluorescence staining (Fig. 6A and Fig. S2F). GCs were treated with miR-484 mimic in the presence of recombinant YAP1 or vehicle control, and overexpression of YAP1 reversed the miR-484-induced reduction of cell viability and proliferation ability (Fig. 6B). Additionally, exogenous overexpression of YAP1 significantly diminished miR-484-induced G0/G1-phase arrest (Fig. 6C). As expected, YAP1 ameliorated the oxidative stress caused by transfection with miR-484 mimic, as evidenced by reduced mtROS levels (Fig. 6D). Moreover, overexpressing YAP1 reversed the aggravation of mitochondrial damage due to miR-484-overexpression in GCs, as demonstrated by the higher ATP content and mtDNA levels (Figs. 6E and 6F). Furthermore, YAP1 markedly inhibited MMP depolarization (Fig. 6G), and rescued GC apoptosis caused by miR-484 mimic transfection (Fig. 6H). These data directly support the notion that YAP1 mRNA is a downstream target of miR-484 that exerts beneficial effects on GC function by modulating mitochondrial pathways and inhibiting mitochondria-dependent apoptosis.

## Discussion

Normal proliferation and differentiation of GCs is an important part of follicular development 24, 25. During follicular development, the precise interaction between oocytes and surrounding GCs is crucial for the generation of fertilizable oocytes and the regulation of ovarian function 26, 27. Therefore, the decline of oocyte competence of women with DOR may arise from abnormal regulation by GCs 28. Studies have shown that miRNAs regulate many biological processes, including cell proliferation, apoptosis, and differentiation. Indeed, there is increasing evidence that miRNAs play critical roles in regulating the function of GCs 29-31. Differential expression and dysregulation of miRNAs is associated with a variety of ovarian disorders 32. For example, miR-22-3p, which was found to be less abundant in the blood of POF patients, has been implicated in apoptotic processes 29. Moreover, Yang et al. demonstrated that miR-23a is upregulated in women with POF and increases human GC apoptosis by suppressing the X-linked inhibitor of apoptosis 18.

Therefore, we characterized miR-484, analyzed its expression levels in different tissues and localization in different cell types, and found that it was highly conserved among different species. The expression level of miR-484 in human GCs from follicular fluid was negatively correlated with ovarian function, with higher expression levels in the DOR group. AMH is expressed in GCs of pre-antral follicles and antral follicles 33. The serum concentration of AMH was found to be proportional to the number of follicles developed in the ovary 34. Currently, AMH is recognized as the gold standard for ovarian function, and the antral follicle count (AFC) also reflects the ovarian reserve in fertile women 35, 36. Here, we found that the expression level of miR-484 in GCs from the follicular fluid of infertile women was negatively correlated with serum levels of AMH and AFC. These results suggested that miR-484 may be closely related to the ovarian reserve, but the causal relationship and the mode of action are still unknown and need to be further explored.

Moreover, our results showed that the expression level of miR-484 in human follicular GCs was negatively correlated with the number of retrieved oocytes and the ratio of high-quality embryos. In patients with high expression levels of miR-484 in GCs, the number of retrieved oocytes and ratio of high-quality embryos were lower than in those with low miR-484 expression levels. Therefore, more studies are needed to clarify the association between the levels of miR-484 and the clinical pregnancy outcomes in ART.

**Figure 7:**
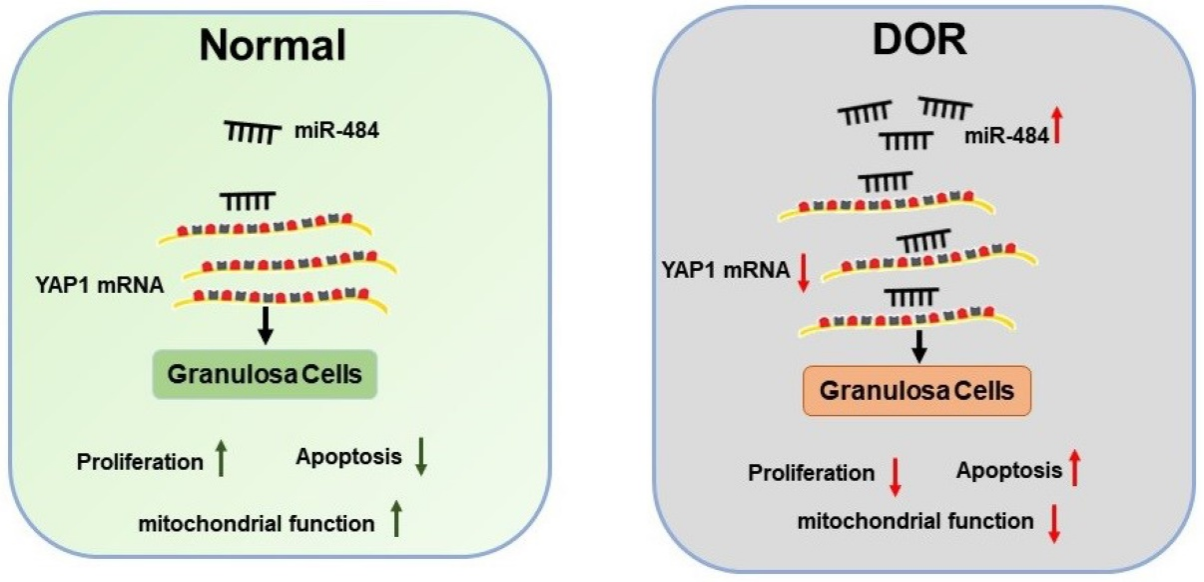
** Schematic diagram demonstrating the molecular mechanisms underlying miR-484 in DOR.** Mir-484 contributes to diminished ovarian reserve by regulating granulosa cell function via YAP1-mediated mitochondrial function and apoptosis.

Further experiments revealed that miR-484 compromised GC function by affecting mitochondrial function and inducing apoptosis. The underlying mechanisms could be attributed to the possible role of miR-484 in regulating mitochondrial function and the mitochondrial apoptotic pathway. By using RNA-seq screening, bioinformatics prediction, qPCR, western blot analysis, and luciferase reporter assays, YAP1 was identified as a direct target of miR-484 in GCs. YAP1 is the main effector of the hippo pathway, which has been reported to play critical roles in regulating biological processes such as cell proliferation, differentiation and aging 37-39. It is believed that YAP1 is expressed in ovarian granulosa cells during follicle development. A graphic illustration of the vital role of miR-484 and YAP1 in DOR is depicted in Fig. 7. Pharmacological inhibition of YAP1 activity disrupted mouse ovarian follicle development *in vitro* and *in vivo*. A Foxl2 promoter-driven knockout of Yap1 in ovarian granulosa cells resulted in increased apoptosis of granulosa cells, decreased the number of corpora lutea, reduced ovarian size, and resulted in subfertility of transgenic mice 40. The relationship between miR-484 and YAP1 has been previously reported. Xu et al. found that miR-484 directly targets the mRNA of YAP1 to inhibit cell viability, promoting the inflammatory response and apoptosis in LPS-treated cardiomyocytes 41. Consistent with these observations, our data showed that si-YAP1 treatment decreased proliferation and induced apoptosis in GCs. Additionally, overexpression of YAP1 offset the negative effects of miR-484 overexpression in GCs by regulating mitochondrial function and apoptosis. Our results suggest that the upregulation of miR-484 may contribute to the pathogenesis of DOR by promoting the apoptosis of GCs via the downregulation of YAP1 expression. This study provides insights into the molecular mechanisms contributing to DOR and may be relevant to ovarian aging or other ovarian disorders.

The miRNA-mRNA regulatory network plays an important role in the development of ovarian diseases, and it was reported that many miRNAs may contribute to DOR by regulating target genes 42, 43. In spite of its promising findings, this study still has some limitations. DOR is the early stage ovarian reserve impairment that may take several years to develop into premature ovarian insufficiency (POI), and subtle alterations may have broader significance during its development 44. Small changes of gene expression must therefore also be analyzed. Further studies focusing on the implications of miRNAs in the pathogenesis of DOR are warranted.

## Supplementary Material

Supplementary figures and table.

## Figures and Tables

**Table 1:** Demographic and clinical characteristics of the enrolled participants

Characteristic	NOR group (≤35) (n = 65)	NOR group (> 35) (n = 20)	DOR group (n = 29)
Age (years)	29.3±3.3	38.1±2.1	34.2±4.1*
Infertility duration (years)	3.2±2.3	5.3±3.7	3.6±3.0
Body mass index (kg/m^2^)	22.9±3.4	21.8±2.7	22.5±3.0
FSH (mIU/ml)	7.0±1.5	7.3±2.2	12.2±6.1*
LH (mIU/ml)	4.8±1.7	4.4±2.3	4.6±3.0
Estradiol (pg/ml)	46.1±32.2	46.6±30.5	58.0±50.6
Progesterone (ng/ml)	0.3±0.2	0.4±0.3	0.8±1.8
AMH (ng/ml)	4.9±2.0	3.2±1.5	0.5±0.3*
AFC (number)	23.5±7.7	15.8±5.9	5.0±1.3*
**Sperm source (number)**			
Husband	48	13	22
Donor	17	7	7

NOR, normal ovarian reserve; DOR, diminished ovarian reserve; AFC, antral follicle count; *P < 0.05.
