# Mir-484 contributes to diminished ovarian reserve by regulating granulosa cell function via YAP1-mediated mitochondrial function and apoptosis: Erratum

**DOI:** 10.7150/ijbs.84828

**Published:** 2023-06-21

**Authors:** Huiying Li, Xiaofei Wang, Hongbei Mu, Qiaojuan Mei, Yu Liu, Zou Min, Ling Zhang, Ping Su, Wenpei Xiang

**Affiliations:** 1 Institute of Reproductive Health, Tongji Medical College, Huazhong University of Science and Technology, Wuhan, Hubei 430030, China.; 2 Center of Reproductive Medicine, Tongji Medical College, Huazhong University of Science and Technology, Wuhan, Hubei 430030, China.

During preparation of the figures of this paper, we made an error in the Figure 2C and Figure 2F. We have now corrected this error by replacing the incorrect image with the correct one in the new Figure 2. We want to clarify that this mistake was made unintentionally and does not impact the conclusions drawn in our article. Please refer to the corrected Figure 2 below as an erratum. We apologize for any inconvenience that may have been caused.

## Figures and Tables

**Figure 2:**
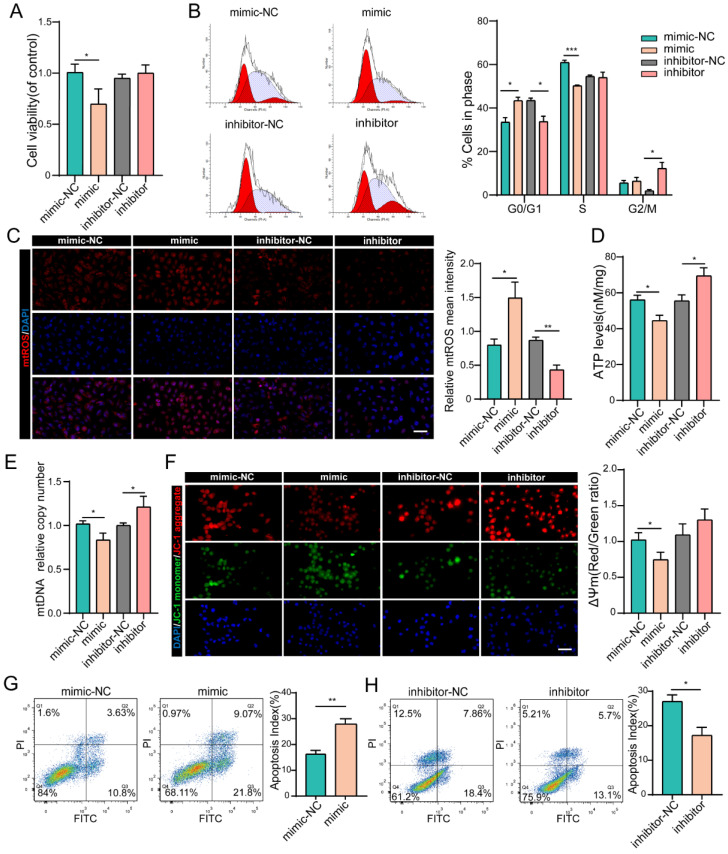
Correct image.

