# Supplementary material for: Mir-484 contributes to diminished ovarian reserve by regulating granulosa cell function via YAP1-mediated mitochondrial function and apoptosis
Source: Int J Biol Sci. 2022 Jan 1;18(3):1008–21. doi: 10.7150/ijbs.68028 (PMC8771835; doi:10.7150/ijbs.68028)
Supplement: Supplementary file 1 — Supplementary figures and table. [file ijbsv18p1008s1.pdf]

Supplementary materials

Fig.S1

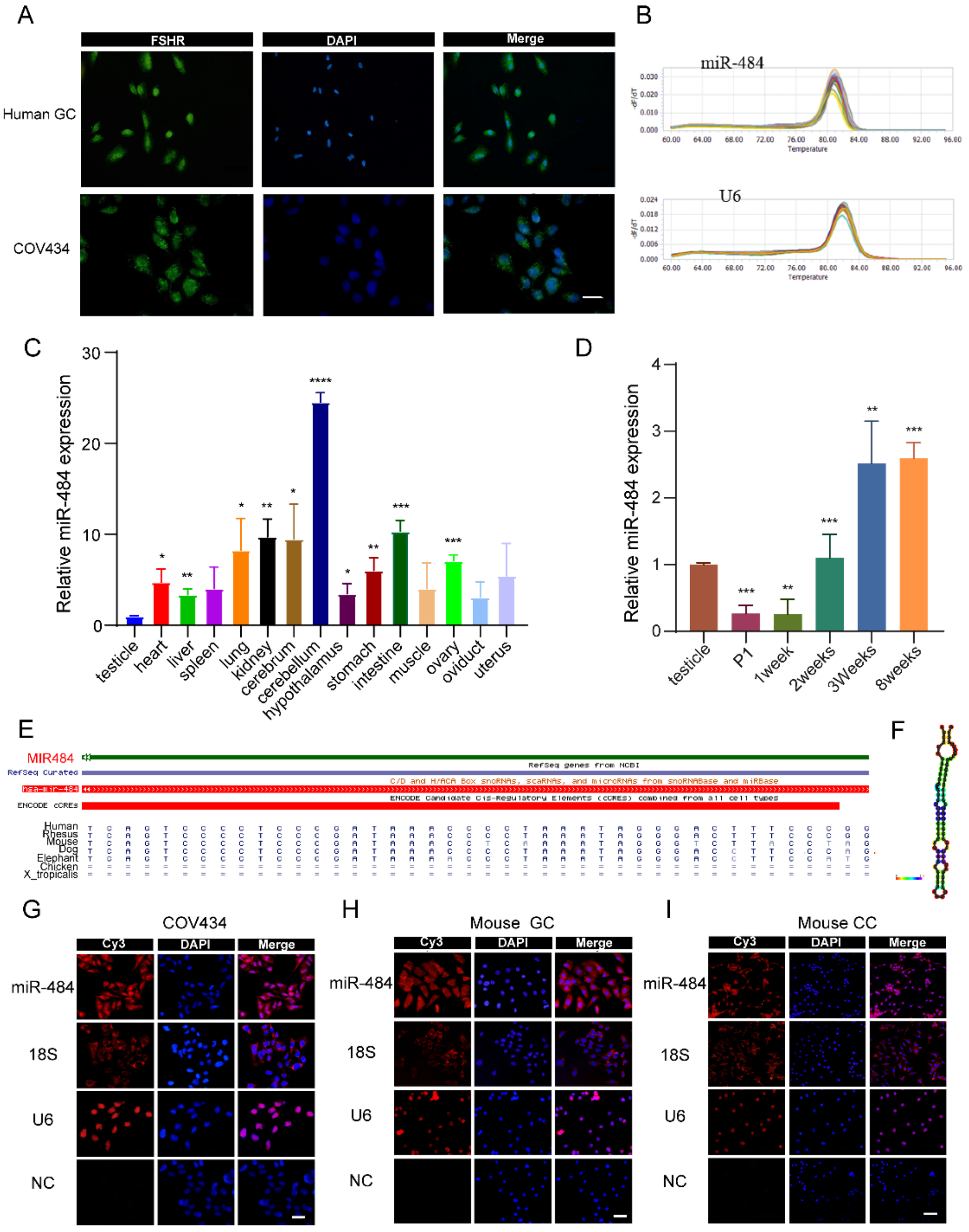

Fig.S2

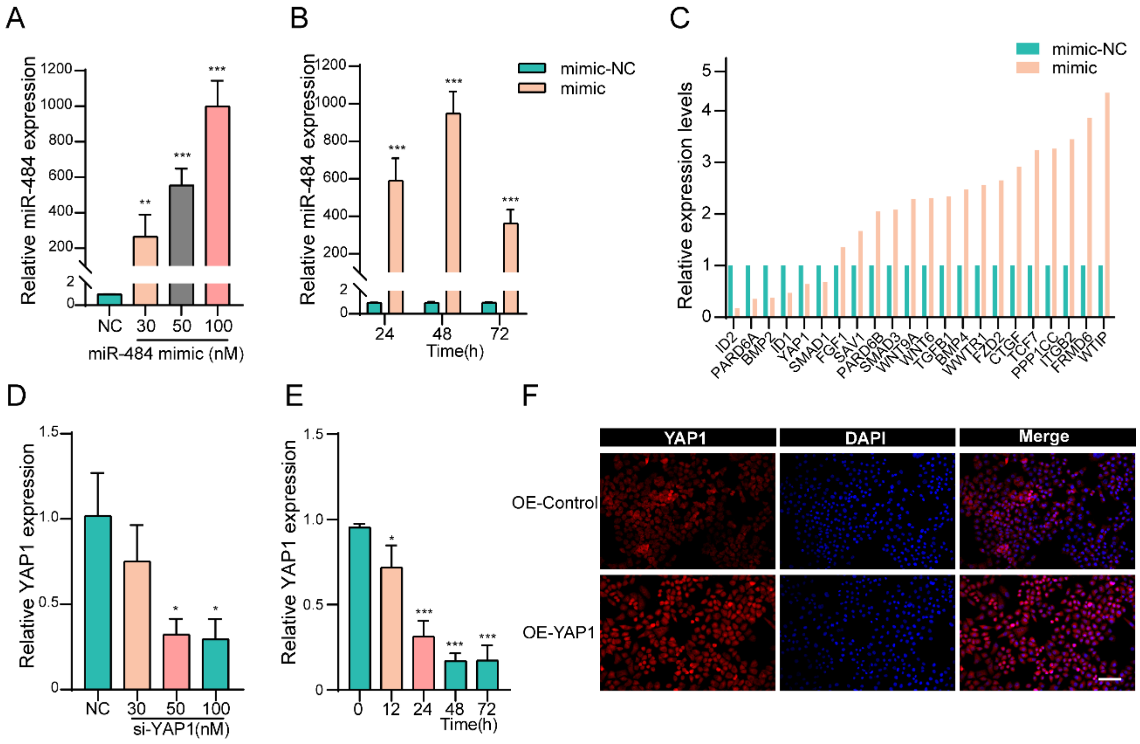

A
Relative miR-484 expression
NC
30
50
100
miR-484 mimic (nM)
B
mimic-NC
mimic
Relative miR-484 expression
Time(h)
C
mimic-NC
mimic
Relative expression levels
ID2
PARD6A
BMP2
ID1
YAP1
SMAD1
FGF1
SAV1
PARD6B
SMAD3
WNT9A
WNT6
TGFB1
BMP4
WWTR1
FZD2
CTGF
TCF7
PPP1CC
ITGB2
FRMD6
WTIP
D
Relative YAP1 expression
si-YAP1(nM)
E
Relative YAP1 expression
Time(h)
F
YAP1
DAPI
Merge
OE-Control
OE-YAP1

Supplementary table 1. Primer sequences used in this study.

Primer name	Sequence
*U6*_F	CTCGCTTCGGCAGCACA
*U6*_R	AACGCTTCACGAATTTGCGT
*miR-484*_F	GCGTCAGGCTCAGTCCCCT
*miR-484*_R	AGTGCAGGGTCCGAGGTATT
*YAP1*_F	TAGCCCTGCGTAGCCAGTTA
*YAP1*_R	TCATGCTTAGTCCACTGTCTGT
*β-actin*_F	CCTTCCTGGGCATGGAGTC
*β-actin*_R	TGATCTTCATTGTGCTGGGTG
*ND-1*_F	CCCTAAAACCCGCCACATCT
*ND-1*_R	GAGCGATGGTGAGAGCTAAGGT
*β-globin*_F	AAAGGTGCCCTTGAGGTTGTC
*β-globin*_R	TGAAGGCTCATGGCAAGAAA
